# Interfacial Atomic Structure of Twisted Few-Layer Graphene

**DOI:** 10.1038/srep21273

**Published:** 2016-02-18

**Authors:** Ryo Ishikawa, Nathan R. Lugg, Kazutoshi Inoue, Hidetaka Sawada, Takashi Taniguchi, Naoya Shibata, Yuichi Ikuhara

**Affiliations:** 1 Institute of Engineering Innovation, University of Tokyo, Bunkyo, Tokyo 113-8656, Japan; 2 World Premier International Research Center, Advanced Institute for Materials Research, Tohoku University, Sendai, 980-8577, Japan; 3 Electron Optics Division, JEOL Ltd., Tokyo 196-8558, Japan; 4 Advanced Key Technologies Division, National Institute for Materials Science, Tsukuba 305-0044, Japan

## Abstract

A twist in bi- or few-layer graphene breaks the local symmetry, introducing a number of intriguing physical properties such as opening new bandgaps. Therefore, determining the twisted atomic structure is critical to understanding and controlling the functional properties of graphene. Combining low-angle annular dark-field electron microscopy with image simulations, we directly determine the atomic structure of twisted few-layer graphene in terms of a moiré superstructure which is parameterized by a single twist angle and lattice constant. This method is shown to be a powerful tool for accurately determining the atomic structure of two-dimensional materials such as graphene, even in the presence of experimental errors. Using coincidence-site-lattice and displacement-shift-complete theories, we show that the *in-plane* translation state between layers is not a significant structure parameter, explaining why the present method is adequate not only for bilayer graphene but also a few-layered twisted graphene.

Since the discovery of monolayer graphene^1^, it has become apparent that the general class of two-dimensional materials such as MoS_2_, black phosphorus and *h*-BN possess a wide variety of physical properties for use in optoelectronics, magnetism, electronic transportation and beyond^2^,^3^,^4^. While monolayer graphene has excellent charge carrier mobility^5^,^6^, it has a zero-bandgap due to its semi-metallic nature, posing a challenge for its use in transistor applications. A number of attempts have been made to obtain a non-zero bandgap for graphene: impurity doping, molecular adsorption on the surface, graphene nanoribbon^7^ fabrication and bilayer graphene (BG) combined with external electric fields^8^,^9^. The bandgap of graphene nanoribbon has a strong dependence of the edge atomic structures such as Klein, zigzag and armchair configurations^10^, similar to the way in which the chirality of a carbon nanotube affects its electronic properties. Symmetry breaking is another option when considering how to open a bandgap in graphene since the electronic structure of BG is exceptionally sensitive to the lattice symmetry. In such a situation, an applied external field breaks the inversion symmetry between interlayers in BG and consequently a bandgap is opened. The same mechanism is valid for the twisted bilayer graphene (TBG), which can also potentially acquire a finite bandgap^11^.

Monolayer graphene consists of two inequivalent sublattices indexed as A and B, shown in Fig. 1(a). Inversion symmetric AB-stacking bilayer graphene has a zero-bandgap. If the two layers are aligned along the *c*-axis (AA-stacking), the inversion symmetry is broken and the band structure obtains a non-zero bandgap. Unfortunately, however, the AA-stacking structure is energetically unfavorable as compared with AB-stacking and therefore the AA-stacking structure is difficult to form via a simple translation. But, due to the weakness of van der Waals force between interlayers, BG has three degrees of freedom: a two-dimensional relative ‘*in-plane*’ translation ***T*** and a relative rotation *θ*. While a simple translation cannot easily form the AA-stacking structure, a relative rotation between layers can provide a new class of moiré superstructure which contains a large amount of AA-stacking and exhibits a non-zero bandgap^11^. This unique band topology has a strong correlation to the atomic structure and it is therefore essential to establish a robust method for the determination of interfacial atomic structure of moiré superstructures.

Recently, low voltage scanning transmission electron microscope (STEM) has become a versatile tool for the atomic structure analysis in two-dimensional materials and it becomes possible to observe individual defect atoms^12^. Furthermore, the local electronic and chemical bonding states such single atom defects has been identified through electron energy-loss spectroscopy^13^. To date, the interfacial atomic structure of TBG has been mainly investigated by reciprocal-space electron microscopy methods–for instance, by selected-area electron diffraction or Fourier spectrum analysis of atomic-resolution S/TEM images^14^. However, these reports have focused on the measuring only the twist angle between layers of TBG since the measurement accuracy is insufficient to directly determine the atomic structures of TBG.

In this study we show how to overcome these insufficiencies and determine the interfacial atomic structure of TBG, by using a combination of coincidence-site-lattice (CSL) and displacement-shift-complete (DSC) theories^15^,^16^ with low-angle annular dark-field (LAADF) STEM imaging, where we use a moiré superstructure parameterized by a relative twist angle and a lattice constant. Once the interfacial atomic structure of TBG is identified, it becomes possible to calculate the fraction of locally formed (pseudo) AA-stacking regions and their spatial distribution, based on DSC theory. Misorientation between layers in van der Waals heterostructures is inevitable and therefore the present method would be useful for the general determination of such interfacial atomic structures^17^.

TBG moiré superstructures have been mathematically investigated based on the Diophantine equation^18^. The same result can be derived from CSL theory which has been used to describe grain boundary structures^15^,^19^. Here we define the notation for TBG moiré superstructures. Figure 1(a) shows an atomic-resolution LAADF image obtained from monolayer graphene with basis translational vectors defined as:


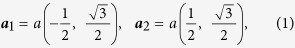


where the *a* is the lattice constant (2.46  Å for graphene). An arbitrary translation vector for the lattice is given by τ(*m*, *n*) = *m**a***_1_ + *n**a***_2_ + δ, where δ is **0** or (***a***_1_ + ***a***_2_)/3 for sublattice A or B, respectively. For simplicity, we begin by considering AA-stacking bilayer graphene and later we will discuss the structure with other in-plane translation states. When a graphene layer in BG is rotated along the [0001] direction (perpendicular to Fig. 1(a)) relative to the other layer without any in-plane relative translations, coincidence sites appear for certain angles and a TBG moiré superstructure is formed. Taking into account the hexagonal symmetry, the twist angle *θ* satisfies the sublattice coincidence condition: τ(*n*, *m*) = **R**(*θ*)τ(*m*, *n*) (where **R** is a rotation matrix). The moiré superstructure angle is therefore given by


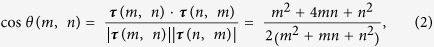


where *m* and *n* are coprime positive integers. In words: a moiré superstructure is formed when, after rotating the sublattice A by angle *θ*, the rotated lattice point (*m*, *n*) aligns with a point on the unrotated sublattice. In a practical sense, if we have two graphene layers (assumed to be AA stacking originally), then a moiré superstructure is formed by rotating one relative to the other. After the rotation, the carbon atom originally at the point (*m*, *n*), is still AA-stacking in its new (rotated) location. Under this condition, the TBG moiré superstructure consists of primitive moiré lattice vectors (τ_1_ τ_2_)^*t*^ = ***M*** (***a***_1_ ***a***_2_)^*t*^ (the superscript *t* means transpose of the matrix) and lattice constant *L*(*m*, *n*) is defined by


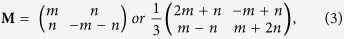



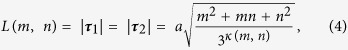



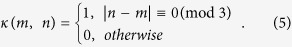


We define the moiré lattice vectors for a basic (or an unrotated) monolayer graphene and, for simplicity, we use the condition *m* < *n*. The matrix **M** as defined above is for κ(*m*, *n*) = 0. For the case where κ(*m*, *n*) = 1, the primitive unit cell is reducible from the dotted-parallelogram (first matrix in Eq. (3)) to the solid-parallelogram (second matrix in Eq. (2)) as shown in Fig. 1(c), and the lattice constant is 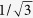 more compact. A dimensionless parameter is more useful to describe the structure and therefore we use the planar coincidence index ∑(*m*, *n*) (*m* < *n*): the area-ratio between moiré superstructure and graphene primitive unit cells, defined by


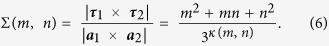


Figure 1(b,c) show simulated LAADF images for ∑(2,3) = 19, and ∑(1,7) = 19. Although these have the same conjugate twisted angles 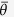
(see Eq. (7)), lattice parameters and coincidence indices, their local structures are distinct–e.g., they have local hexagonal or triangular symmetries in projection respectively–hence direct observation has great potential to distinguish between different TBG atomic structures.

In the context of a moiré superstructure, we have used a twist angle and lattice constant to systematically investigate the possible structures of TBG (see some specific examples for values of ∑  ≤ 139 in Supplementary Information). Figure 2(a) shows the twist angle as a function of a pairs of (*m, n*) (where *m* < *n* ≤ 64); the twist angle has almost linear dependence on the ratio *m*/*n*, where we ignore the pairs of (*m*, *n*) with gcd(*m*, *n*) ≠ 1 (gcd: the greatest common divisor). Due to the symmetries of graphene, a twist of either *θ* or π/3-*θ* will produce the same diffraction pattern (see Supplementary Information). The conjugate twist angle 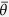 is defined as the minimum of these two:


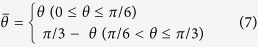


such that *θ* + 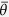  = π/3. The conjugate twist angle map is shown in Fig. 2(b). The other unique structure parameter of a moiré superstructure, the moiré lattice constant, is shown in Fig. 2(c) and it has an ellipsoidal dependence on a pair of (*m, n*), evident from Eq. (4). Since the twist angle and moiré lattice constant vary differently with (*m*, *n*), these structural parameters can be used to significantly narrow down the possible pairs (*m*, *n*), which uniquely define a moiré superstructure. While state-of-the-art STEM can produce sub-angstrom precision measurements^20^, this is usually over a very small field-of-view; over a large field-of-view, errors in precision are compounded resulting in typical errors in the range of ~1° and ~0.1 nm for the measurement of twist angle and the length, respectively. Several methods have been used to measure the twist angles^21^,^22^, but there has not been much success in to determining both the twist angle and the moiré lattice constant using these techniques, suggesting that S/TEM has some advantages for the determination of the structure. To demonstrate the utility of the proposed method for the determination of a TBG structure, we selected the condition of 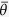 = 10 ± 1° and *L* = 5.0 ± 0.2 nm as an example, which include relatively large experimental errors. The indices (*m*, *n*) corresponding to these values are highlighted by dark green or blue and cyan (conjugate twist angle and moiré lattice constant respectively) in Fig. 2(d). In this case, even including errors, there are only two moiré superstructure candidates that match these conditions with indices (*m*, *n*) = (2, 19) or (17, 23), where the two structures have the same structure properties ∑ = 403, *L* = 4.93 nm and 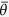 = 9.89°. Such twist angles and the moiré lattice parameters of TBG can be measureable by atomic-resolution electron microscopy, including Fourier analysis. For more accurate structural determination, direct comparison between an experimental atomic-resolution STEM and image simulation is required.

For a full structural analysis, here we consider the ‘*in-plane*’ translation state between layers based on displacement-shift-complete (DSC) theory, which we will describe shortly. In a similar manner to graphite, the interlayer interaction energy for bilayer graphene is minimized and maximized at AB- and AA-stacking, respectively^23^. Another possible translation state is SP-stacking (SP: saddle point), where the Peierls potential along the armchair direction is minimized and may possibly be formed, for instance, during the mechanical process of exfoliation^24^,^25^,^26^. The models for AA-, AB- and SP-stacking are given in Fig. 3(a). For the case of graphite (or untwisted multi-layer graphene), the in-plane displacement between AA- and AB-stacking orders is fairly large of 1.42 Å, and this displacement can therefore be used to define and identify the stacking mode. However, for the twisted case we will see that the displacement between the two stacking orders becomes significantly reduced as a function of coincidence index, and the in-plane translation between layers is no longer an adequate parameter to describe the structure.

To investigate initial/local stacking order in TBG, we selected ∑(6,7) = 127 TBG structure, initially having AA-stacking before twisting, as an example (the corresponding experimental result will be discussed later). Figure 3(b) shows the projected atomic structure (red and blue lattice dichromatic pattern) and the solid parallelogram shows the primitive unit cell. The arrows on the blue-layer show the moiré lattice vector of τ_1_ = 6**a**_1_ + 7**a**_2_. As indicated by the labeled colored circles, the structure consists of small patches of the locally formed pseudo AA-, AB-, and SP-stacking regions. Even though the in-plane translation state of TBG was initially AA-stacking before being twisted by a relative rotation, the fraction of local AA-stacking is significantly suppressed afterwards. The initial stacking order is therefore no longer a reasonable parameter to describe the moiré superstructure. Instead, we will consider what insight is offered by the local stacking order between nearest-neighbors. When we translate a blue lattice point onto a red lattice point on the same sublattice (each layer has two sublattices) by a translation vector ***T***, we recreate the identical moiré superstructure, which we call DSC translation vector. To find all the inequivalent DSC translation vectors, we explore the nearest-neighbor translation vectors, from red to blue lattices. Figure 3(c) shows the nearest-neighbor DSC translation vectors for ∑(6,7) = 127 TBG, where the vectors have the form of a hexagonal arrangement of anticlockwise vortices. From the DCS translation vector map, we see that pseudo AA-, AB- and SP-stacking regions are locally formed at the core, ridge and triple junction of vortices, respectively. The set of DSC translation vectors forms a lattice known as a DSC lattice and is shown in Fig. 3(d). The position of a point on the DSC lattice corresponds to the magnitude and direction of a DSC translation vector and the origin is the graphene sublattice A (labeled AA in Fig. 3(d)). The set of inequivalent translation vectors forms the so-called reduced DSC lattice (which contains ∑ points) and the basis of the reduced DSC lattice is given by


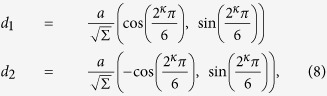


where κ is given in Eq. (5). For the case shown in Fig. 3(d), we consider initial AA-stacking, hence the origin, vertices, and edge of the hexagon with labeled AB in Fig. 3(d) correspond to the initial AA-, AB- and SP-stacking translation vectors, respectively.

An in-plane displacement along any DSC lattice translation vector will recreate the CSL, thus any variation of in-plane displacement will fall within the Wigner-Seitz (WS) cell of the DSC lattice (see the small hexagonal cell in Fig. 3(d)). The largest possible in-plane displacement is thus 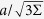 (the distance from the center to the vertex of WS-cell), and hence the displacement decreases as a function of coincidence index ∑. According to the DSC lattice map in Fig. 3(d), the minimum global displacement from AA- to AB-stacking corresponds to the smallest vector between AB-stacking position and the nearest DSC lattice, as indicated by the vector ***d***_AB_. Using Eq. (8), we explore the minimum displacement lengths from AA- to AB- or SP-stacking (***d***_AB_, ***d***_SP_ and the side of the WS-cell respectively) which are plotted as a function of coincidence index in Fig. 3(e) (for ∑ ≤ 397). Compared to the untwisted case (1.42 Å), the displacement lengths of ***d***_AB_, ***d***_SP_ are almost all less than 0.1 Å, which is negligibly small. Therefore, we conclude that, for the determination of moiré superstructure, it is reasonable to ignore in-plane translational between layers, particularly at higher coincidence indices. This result is significant because it shows that it is possible to determine the single interfacial atomic structure of not only bilayer graphene but also a few layer graphene.

Once we know the DSC lattice, it is possible to estimate the fraction of locally formed pseudo-stacking regions by counting the number of DSC lattice points within a critical distance from the initial AA-, AB- or SP-stacking positions. Here we use the following criterion for the pseudo-stacking (to avoid repetition): the magnitude of a translation vector measured from the stacking positions is less than *r*_0_ = *a*_0_/4 (0.20 Å, 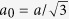), or within a circle shown in Fig. 3(d). With increasing coincidence index, the number of translation vectors in the reduced DSC lattice (within the hexagon labeled by AB in Fig. 3(d)) will increase and hence the fraction of pseudo AA-stacking will converge to the ratio 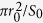 (~7.5%), where S_0_ is the area of the hexagon labeled by AB in Fig. 3(d). We note that the pseudo AB- and SP-stacking are located, respectively, on the vertex and edge of AB-sublattice hexagon, and hence their fractions are, respectively, two and three times higher than that of pseudo AA-stacking. Figure 3(f) shows the fractions of each specific pseudo-stacking and the others as a function of coincidence index, where the horizontal lines are the expected convergence value of the ratio. We note here that the interfacial structure analysis related to the in-plane displacement has not been performed by exploring Diophantine equation; the combination of CSL and DSC theories are advantageous for such analyses since the minimum displacement length and locally formed pseudo-stacking area can be estimated using this technique. Although the atomic structures of each moiré superstructure are very different, intriguingly the fraction of specific pseudo-stacking is almost constant as a function of coincidence index. However, for very lower twist angle cases, local pseudo AA-stacking regions should cover a large area, increasing as a function of coincidence index ∑. This may introduce new intriguing physical properties^3^.

On the basis of the structure modeling in the previous section, we will determine the interfacial atomic structure of few-layer graphene by using atomic-resolution electron microscopy. To observe the atomic structure of few-layer graphene, a spatial resolution higher than required for the case of monolayer graphene, is needed since the overlapping, layered structure will contain features on a finer scale than an individual layer. Here we use an accelerating voltage of 80 kV, which is below the knock-on damage threshold of 86 kV of graphene^27^. In addition, ADF STEM image contrast is also strongly affected by the angular range of the annular detector used. Therefore, to enhance the visibility of the TBG atomic structure, we first determine the optimum inner angle for the ADF detector, via image simulations. Figure 4 shows the simulated (a) LAADF STEM images of ∑(3,7) = 79 TBG, with the AB-(low-angle ADF), (b) MAADF (medium-angle ADF) and (c) HAADF (high-angle ADF)stacking structure model shown in Fig. 4(d). The probe-forming aperture half-angle is 27 mrad (milliradian) and the ADF detectors span (a) 32–240 mrad (LAADF), (b) 45–240 mrad (MAADF) and (c) 64–240 mrad (HAADF). Although there is no significant difference in spatial resolution, the mean intensity of the LAADF image is about four times higher than that of HAADF image (the maximum intensities are (a) 1%, (b) 0.5% and (c) 0.25% of the incident electrons for LAADF, MAADF and HAADF respectively). The inner angle of the LAADF detector is very close to the bright field region; the resultant contrast is strongly affected by the elastic scattering of the probe. Diffraction contrast can enhance the intensity at locally high projection-symmetry area such as AA- or AB-stacking area and also provide better signal level, which can be seen in Fig. 4(b). The amount of elastic scattering collected by the detector decreases with increasing detector inner-angle and hence MAADF and HAADF imaging gradually lose the sensitivity of the local symmetry. Therefore, the LAADF mode has a definite advantage since it collects more signal, which is particularly beneficial under low electron dose illumination conditions. Moreover, the LAADF mode has the largest standard deviation (contrast) of the three imaging modes, suggesting that LAADF imaging is more sensitive to the degree of atomic overlap along the in-plane translation direction. This effect can be clearly seen in the normalized intensity profile along the X and X’ direction, inset in Fig. 4(b): the LAADF image intensity has much stronger column dependence than that of HAADF image (the projected atom distances for the positions labeled X and X’ are 0.16 Å and 0.28 Å respectively). The higher sensitivity of LAADF with regards to the degree of atomic overlap is helpful when exploring the projected local symmetry and structure for TBG. Therefore, we proceed by using the LAADF imaging mode for our experimental analysis.

Figure 5(a,b) show low-magnification HAADF and LAADF STEM images, respectively, obtained from few-layer graphene. The HAADF image was acquired after the LAADF image. The inner angles of the ADF detectors were 32 and 64 mrad for the LAADF and HAADF images respectively. The top-right region is vacuum and the number of graphene layers is given on the HAADF image, estimated simply from the HAADF intensity, since the observed mean intensities increase approximately linearly with the number of graphene layers. We have confirmed that the edge region is monolayer graphene and that the bilayer region is AB-stacking via high-magnification HAADF observations. In the HAADF image, single silicon atoms on the graphene (contamination) are clearly seen as brighter dot contrast (Z-contrast), emphasizing the resolving power of HAADF STEM. However, the moiré pattern is faint and HAADF imaging mode may not be suitable for TBG structure analysis, as discussed previously. On the other hand, in the LAADF image the moiré pattern, with hexagonal-like symmetry, is clearly visible, particularly in the regions with 3 or 4 graphene layers. This demonstrates that the LAADF imaging mode has a high sensitivity to the moiré superstructure and it is therefore more suitable to analyze the atomic structure of twisted graphene. This is in agreement with the simulated results in the previous section.

To determine the moiré superstructure, we will investigate both the twist angle and the lattice constant by using the Discrete Fourier Transform (DFT) spectrum of the LAADF image, shown in Fig. 5(c). As indicated by arrowheads, the Fourier spots, corresponding to the 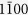 Bragg reflection (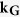), are split along the azimuth direction; the angle between these spots is the conjugate twist angle. The Fourier spots are fitted with 2D Gaussian profiles and the conjugate twist angle between a pair of Fourier spots is measured to be 5.2 ± 0.3°. Around the direct spot (and other reflections), new Fourier spots are clearly visible (marked by the arrows in Fig. 5(c)). These satellite spots, defined by reciprocal space vector 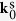, should be related to the correlation length of moiré pattern. It is noteworthy that DFT spectrum is inequivalent to electron diffraction; the satellite spots do not appear in electron diffraction pattern. In diffraction experiments, the two layers produce the same diffraction pattern and the information of moiré lattice constant is lost (see Supplementary Information). However, in Fourier analysis, the moiré superstructure–which is a projection of the two layers–is directly recorded as a LAADF STEM image and it becomes possible to extract the information of moiré lattice constant by using the satellite spots. Therefore, for the determination of moiré superstructure, it is required to obtain atomic-resolution images of bilayer graphene. According to O-lattice theory^15^,^16^. for a small twist angle (typically less than 10°), highly ordered areas (or AA-stacking areas) are periodically arranged, forming a moiré superstructure in which the symmetry of the ordered areas retains the original symmetry of monolayer graphene. Therefore since we know the lattice parameter of monolayer graphene, we can estimate the lattice parameter of moiré superstructure from the ratio 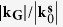
which gives a value of 2.71 ± 0.04 nm. The lattice constant is also directly measureable from atomic-resolution LAADF image of Fig. 5(d) and the distance X-X’ is determined to be ~2.7 nm, which is in agreement with the DFT spectrum analysis measurement. According to the LAADF image of Fig. 5(b), the moiré pattern appears in all the regions with more than three-layer graphene (i.e., not the single and bilayer regions). It is therefore reasonable to conclude that the top (or bottom) few layers are rotated relative to the bottom (or top) AB-stacking bilayer graphene, and only a single twist interface exists in the few-layer graphene, as shown in the model of Fig. 5(e). We note that the moiré pattern in the top-left region of Fig. 5(b) is spreading slightly, which may originate from the modulation of the local stacking order. However, as we have discussed with DSC theory (in regard to Fig. 3(e)), the moiré pattern should be insensitive to both local stacking faults and the twist interface location, owing to their small DSC vector lengths of around 0.1 Å. On the other hand, one can also see the nanotube-like contrast at the top-left edge region and hence the present few-layer graphene may be formed by folding a piece of bilayer graphene on top of itself. Given the measured twist angle and lattice constant, we explore the candidates for the moiré superstructure and the matching map is shown in Fig. 5(f): the only candidates are (*m*, *n*) = (6, 7) or (1, 19). Although our present spatial resolution is not enough to distinguish between these two structures (particularly for the few-layer case), the structure parameters were successfully determined to be ∑ = 127, *L* = 2.77 nm and 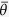 = 5.09°. Simulated LAADF image of four-layer ∑(6, 7) = 127 is embedded on Fig. 5(d), which reproduces the experiment very well and strongly supports the validity of our structure determination.

In summary, we have demonstrated atomic structure determination in twisted few-layer graphene using LAADF STEM imaging in conjunction with CSL and DSC theory. We have shown that such structures can be determined in terms of two parameters only: a conjugate twist angle and a moiré lattice constant. The LAADF STEM measurement precision in these parameters might not be sufficient to directly determine the interfacial atomic structure, however we have overcome these experimental errors with the aid of CSL and DSC theory. This simple method has shown to be a powerful tool for greatly reducing the number of possible moiré superstructures for a given twist angle and lattice parameter. From this reduced set of possible superstructures, the true structure of twisted graphene is determined through atomic-resolution imaging. Such TEM analyses can be routinely performed directly after the measurement of physical properties in scanning probe microscopy or scanning tunneling microscopy, and will provide the essential information to understand the origin of new physical functionalities.

## Methods

Graphite single crystals were prepared by recrystallized graphite crystal at high pressure annealing in 2.5 GPa and 2773 K. Graphite single crystals were gently crushed in ethanol and powers were sonicated in the ultrasonic bath for 60 mins. Subsequently, the powders were dispersed on a perforated amorphous TEM grid. To remove hydrocarbon contamination, the TEM grid was annealed at around 100 °C for 4 hours in clean vacuum and then transferred into the microscope. The atomic-resolution ADF STEM images were acquired with JEM 300CF installed in University of Tokyo, equipped with JEOL COSMO aberration corrector and cold field emission gun, operated at 80 kV. The probe-forming-aperture semi-angle was 27 mrad, and the inner ADF detector semi-angle for LAADF and HAADF STEM imaging modes were 32 and 64 mrad, respectively. To suppress beam damage, low beam currents were used, typically ~20 pA.

The image simulations were performed using a multislice algorithm with the frozen phonon model (TEMSIM package^28^) for an 80 kV probe with an aperture half-angle of 27 mrad. To compensate the spatial incoherence of the microscope^29^,^30^,^31^,^32^. the simulated images were convolved with a Gaussian finite source size of 1.1 Å (full-width-half-maximum).

## Additional Information

**How to cite this article**: Ishikawa, R. *et al.* Interfacial Atomic Structure of Twisted Few-Layer Graphene. *Sci. Rep.* **6**, 21273; doi: 10.1038/srep21273 (2016).

## Supplementary Material

Supplementary Information

## Figures and Tables

**Figure 1:**
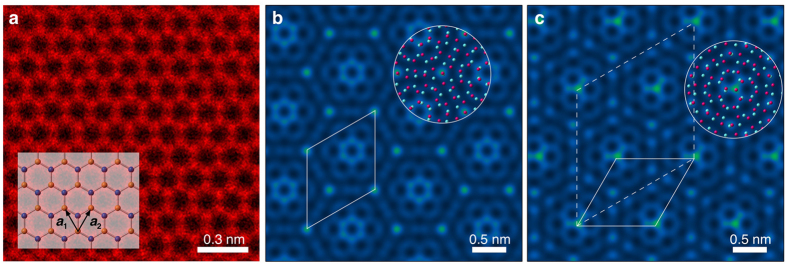
Atomic-resolution LAADF STEM images of monolayer and twisted bilayer graphene. (**a**) Atomic-resolution LAADF STEM image of monolayer graphene. The structure model is overlaid. Blue and yellow lattices show the A and B sublattices. The basis vectors are ***a***_1_ and ***a***_2_. Simulated LAADF STEM images of AA-stacking TBG are shown in (**b**) ∑(2, 3) = 19 (*θ* = 13.17°) and (c) ∑(1, 7) = 19 (*θ* = 46.83°), with the structure models overlaid on the images. The solid parallelograms are the primitive unit cells and the dashed parallelogram is a non-reduced unit cell for the case of *κ*(*m*, *n*) = 1.

**Figure 2:**
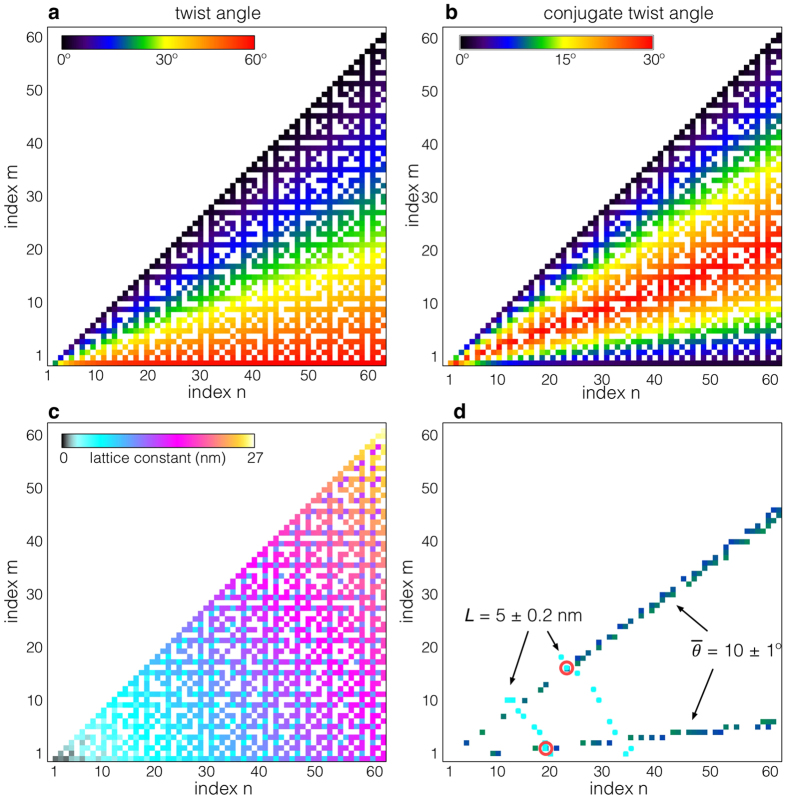
The maps of moiré rotation angle and moiré lattice constant. Maps of (**a**) twisted angle (0 ≤ *θ* ≤ π/3), (**b**) conjugate twisted angle (0 ≤ 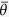 ≤ π/6) and (**c**) moiré lattice constant as functions of (*m*, *n*). (**d**) The two matching candidates for *L* = 5.0 ± 0.2 nm and 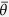 = 10 ± 1° are marked by red circles.

**Figure 3:**
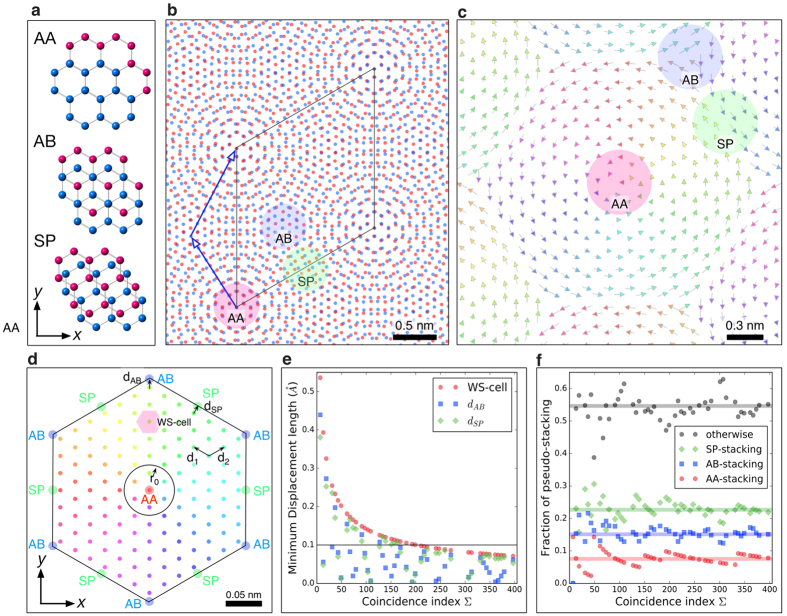
DSC vector map and DSC lattice of ∑127 twisted graphene. (**a**) The structure models of AA-, AB- and SP-stacking-order (blue and red atoms are the respective layers). The translation vector from AA- to AB- or SP-stacking-order is (**a**_1_ + **a**_2_)/3 or **a**_1_/2, respectively. The direction of *x*- and *y*-axis are **a**_1_ + **a**_2_ and **a**_1_ − **a**_2_, respectively. (**b**) The structure model of TBG ∑(6,7) = 127 with AA-stacking. The blue arrows on the blue-layer show the moiré lattice vector of τ_1_ (=6**a**_1_ + 7**a**_2_). The overlaid red, blue and green circles are located on pseudo AA-, AB- and SP-stacking areas, respectively. The blue and red layers are rotated +*θ*/2 or −*θ*/2 from y-axis, respectively (*θ* = 5.09°). (**c**) DCS vectors from red- to blue-lattices in the same coordination of (**a**), where the arrow colors correspond to direction. (**d**) DSC lattice map for TBG ∑(6,7) = 127 in the same coordination of (**a**), where the origin is denoted by AA. The color of DSC lattice indicates the direction of DSC vector as in (**c**). The notation of AB (blue color) and SP (green color) in the large cell indicate the positions of AB and SP stacking order. ***d***_1_, ***d***_2_ are the DSC basic vectors and the WS-cell of the DSC lattice is shown by the small red hexagon, *r*_0_ is defined to be the area containing translation vectors belonging to a specific pseudo-stacking order. (**e**) Minimum displacement shift for AB- and SP-stacking with κ = 0 as a function of coincidence index. (**f**) Fraction of pseudo AA-, AB-, SP- and other-stacking orders as a function of coincidence index, where the horizontal colored lines are the estimated fractions with the criterion of *r*_0_ = *a*_0_/4 (0.20 Å).

**Figure 4:**
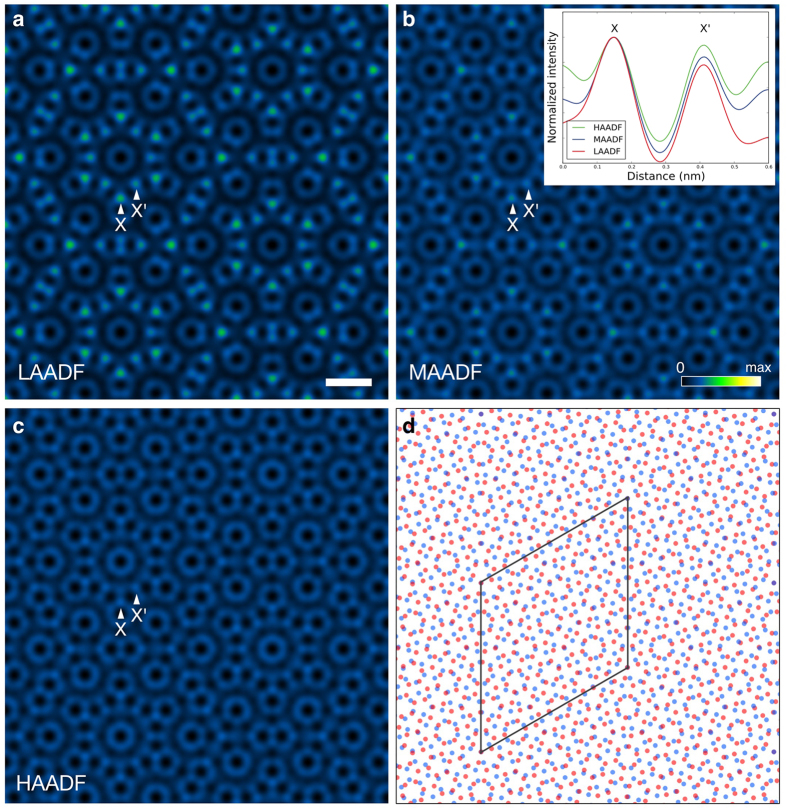
Detector angle dependence of simulated ADF STEM images with ∑79 twisted bilayer graphene. The simulated ADF STEM images of ∑(3,7) = 79 with AB-stacking TBG, where the detector spans (**a**) 32-240 mrad, (**b**) 45–240 mrad and (**c**) 64-240 mrad, respectively. Intensity ranges are 0 to (a) 1% (b) 0.5% and (**c**) 0.25% of the incident electrons. The notations of X and X’ correspond to the locations of small and large hexagonal vertices, respectively. The inset of (**b**) shows the intensity profile along X-X’ direction, where the intensities are normalized to the maximum at X. (**d**) The structure model used for the image simulations, where red and blue atoms correspond to the top and bottom layers respectively, and the parallelogram is the primitive unit cell. The scale bar in (**a**) is 0.5 nm.

**Figure 5:**
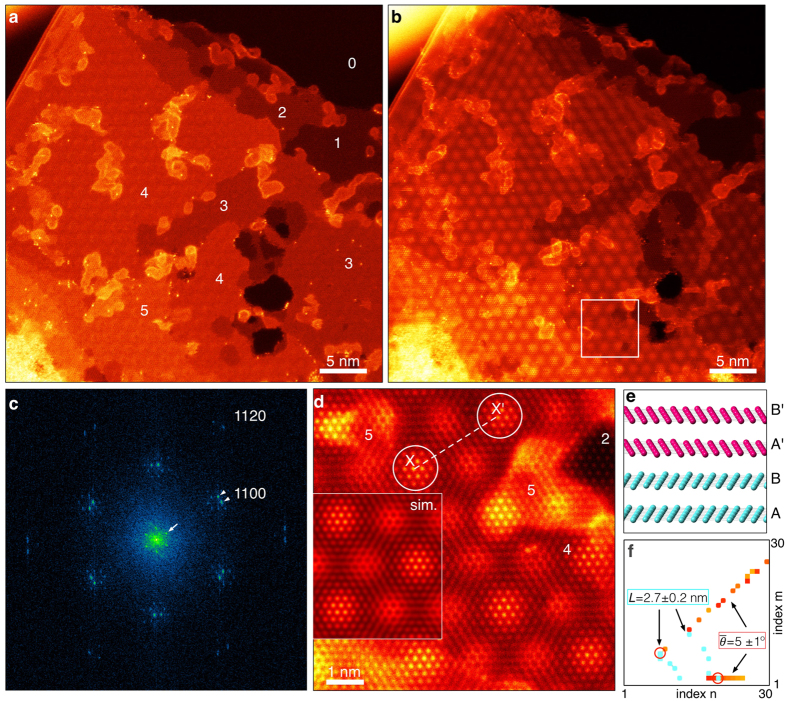
Atomic-resolution ADF STEM image and the Fourier spectrum obtained from few-layer graphene. Atomic-resolution (**a**) HAADF and (**b**) LAADF STEM images obtained from twisted few-layer graphene. (**c**) DFT spectrum obtained from the LAADF image. We have used a Hanning window to remove any artifacts from the discontinuous image boundary condition. (**d**) High-magnification LAADF STEM image of the marked area in (**b**). A simulated LAADF STEM image, with twisted four-layer graphene (AB-stacking) with ∑(6,7) = 127, is overlaid on the left of (**d**). (**e**) Model of the four-layer structure used in the image simulation of (**d**). The top (pink) and bottom (cyan) two-layers have AB stacking order, but the top-layers (noted by **A**′,**B**′) are rotated *θ* = 5.09° relative to the bottom two layers (noted by **A**,**B**). (**f**) The matching candidate as functions of (*m*, *n*), plotted in the same manner as Fig. 2(d).
