# Supplementary material for: Interfacial Atomic Structure of Twisted Few-Layer Graphene
Source: Sci Rep. 2016 Feb 18;6:21273. doi: 10.1038/srep21273 (PMC4758067; doi:10.1038/srep21273)
Supplement: Supplementary Information [file srep21273-s1.pdf]

Supplementary Information

Interfacial Atomic Structure of Twisted Few-Layer Graphene

Ryo Ishikawa[1*], Nathan R. Lugg[1], Kazutoshi Inoue[2], Hidetaka Sawada[3],
Takashi Taniguchi[4], Naoya Shibata[1] and Yuichi Ikuhara[1,2]

[1]Institute of Engineering Innovation, University of Tokyo, Bunkyo, Tokyo 113-8656, Japan

[2]World Premier International Research Center, Advanced Institute for Materials Research, Tohoku University, Sendai, 980-8577, Japan

[3]Electron Optics Division, JEOL Ltd., Tokyo 196-8558, Japan

[4]Advanced Key Technologies Division, National Institute for Materials Science, Tsukuba 305-0044, Japan

*E-mail: ishikawa@sigma.t.u-tokyo.ac.jp

Σ	L (nm)	$\bar{\theta}$	θ	(m, n)
7	0.65	21.79	21.79	(1, 2)
			38.21	(1, 4)
13	0.89	27.8	32.2	(1, 3)
			27.8	(2, 5)
19	1.07	13.17	46.83	(1, 7)
			13.17	(2, 3)
31	1.37	17.9	42.1	(1, 5)
			17.9	(4, 7)
37	1.49	9.43	50.57	(1, 10)
			9.43	(3, 4)
43	1.61	15.18	44.82	(1, 6)
			15.18	(5, 8)
49	1.72	16.43	43.57	(2, 11)
			16.43	(3, 5)
61	1.92	7.34	52.66	(1, 13)
			7.34	(4, 5)
67	2.01	24.43	35.57	(2, 7)
			24.43	(5, 11)
73	2.10	11.64	48.36	(1, 8)
			11.64	(7, 10)
79	2.18	26.01	26.01	(3, 7)
			33.99	(4, 13)
91	2.34	6.01	53.99	(1, 16)
			6.01	(5, 6)
		10.42	49.58	(1, 9)
			10.42	(8, 11)
97	2.42	29.41	29.41	(3, 8)
			30.59	(5, 14)
103	2.49	19.65	40.35	(2, 9)
			19.65	(7, 13)
109	2.56	10.99	49.01	(2, 17)
			10.99	(5, 7)
127	2.77	5.09	54.91	(1, 19)
			5.09	(6, 7)
133	2.83	8.61	51.39	(1, 11)
			8.61	(10, 13)
		25.04	25.04	(4, 9)
			34.96	(5, 17)
139	2.90	25.46	34.54	(3, 10)
			25.46	(7, 16)

Figure S1 | The list of structure parameters for TBG ($\Sigma \leq 139$), where the parameters are coincidence index Σ, lattice parameter L, twist angle θ, conjugate twist angle $\bar{\theta}$ and a pair index (m, n).

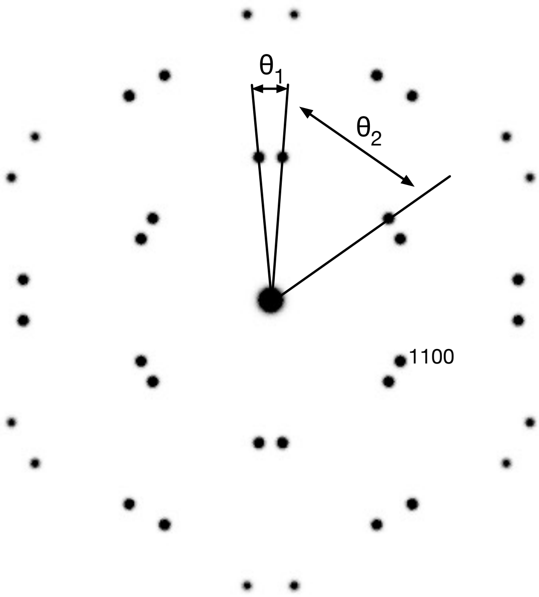

Figure S2 | The electron diffraction pattern calculated from the bilayer graphene $\Sigma(1,10) = 37$, where the true twist angle for $\Sigma(1,10) = 37$ is 50.57 ($=\theta_2$).

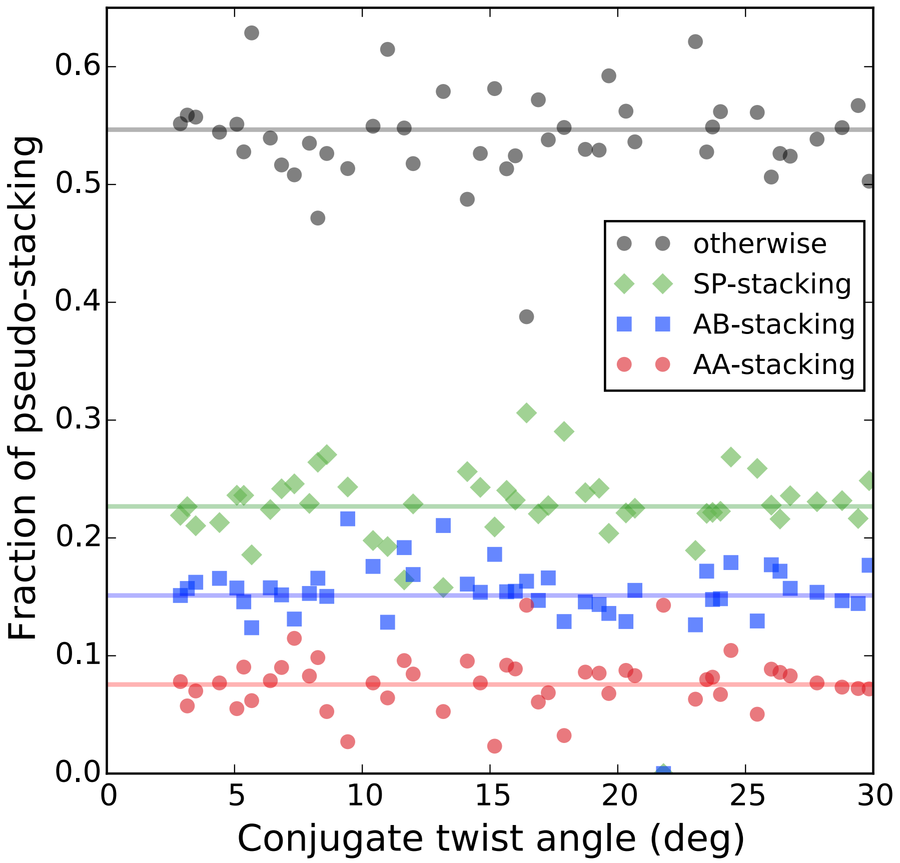

Figure S3 | Fraction of pseudo AA-, AB-, SP- and other-stacking orders as a function of conjugate twist angle. The used angles are calculated from the corresponding coincidence indices (<400) shown in Fig. 3(e). The horizontal colored lines are the estimated fractions with the criterion of $r_0 = a_0/4$ (0.20 Å).
